# Supplementary material for: Cytocompatibility, Antimicrobial and Antioxidant Activity of a Mucoadhesive Biopolymeric Hydrogel Embedding Selenium Nanoparticles Phytosynthesized by Sea Buckthorn Leaf Extract
Source: Pharmaceuticals (Basel). 2023 Dec 22;17(1):23. doi: 10.3390/ph17010023 (PMC10819796; doi:10.3390/ph17010023)
Supplement: Supplementary file 1 [file pharmaceuticals-17-00023-s001.zip › pharmaceuticals-2733165-supplementary.pdf]

Cytocompatibility, Antimicrobial and Antioxidant Activity of a Mucoadhesive Biopolymeric Hydrogel Embedding Selenium Nanoparticles Phyto-Synthesized by Sea Buckthorn Leaf Extract

Naomi Tritean, Luminița Dimitriu, Ștefan-Ovidiu Dima, Rusăndica Stoica, Bogdan Trică, Marius Ghiurea, Ionuț Moraru, Anisoara Cimpean, Florin Oancea and Diana Constantinescu-Aruxandei

Supplementary Information

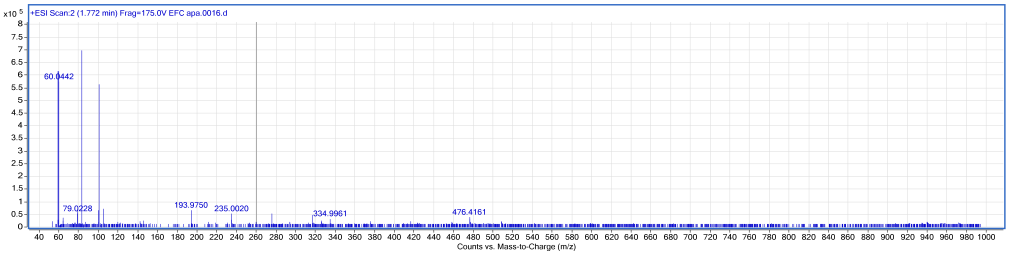

(a)

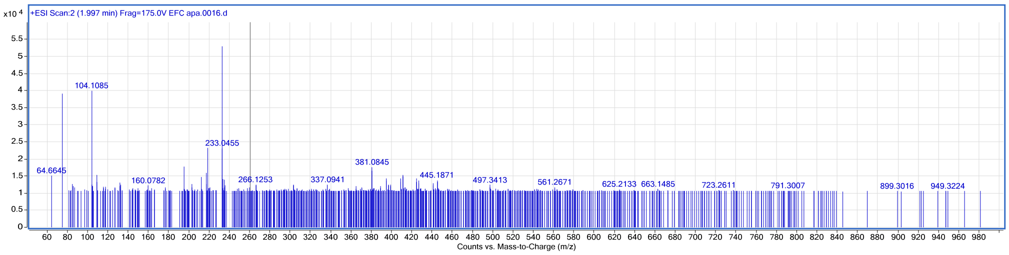

(b)

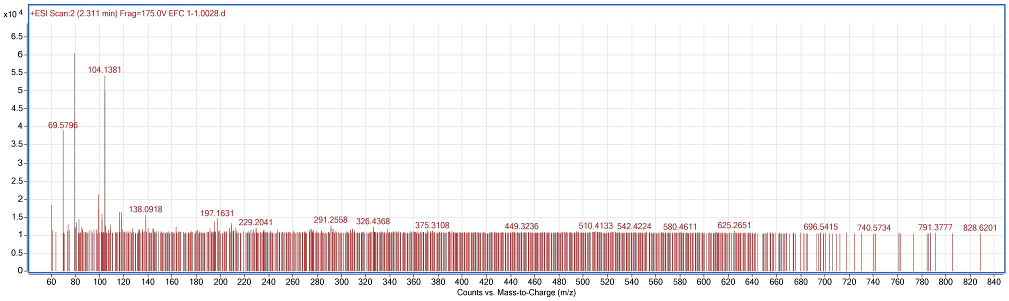

(c)

Figure S1. LC-TOF/MS chromatograms of SbLEx: (**a**)-(**c**) $[M+H]^+$ (m/z).

Table S1. HPLC-DAD linearity results

Compound	λ (nm)	Regression equation	Correlation coefficient
catechin	280	y =6.482x + 1.894	0.9999
epicatechin	280	y =8.102x + 0.904	0.9998
quercetin 3-rutinoside	350	y =14.302x – 0.035	0.9999

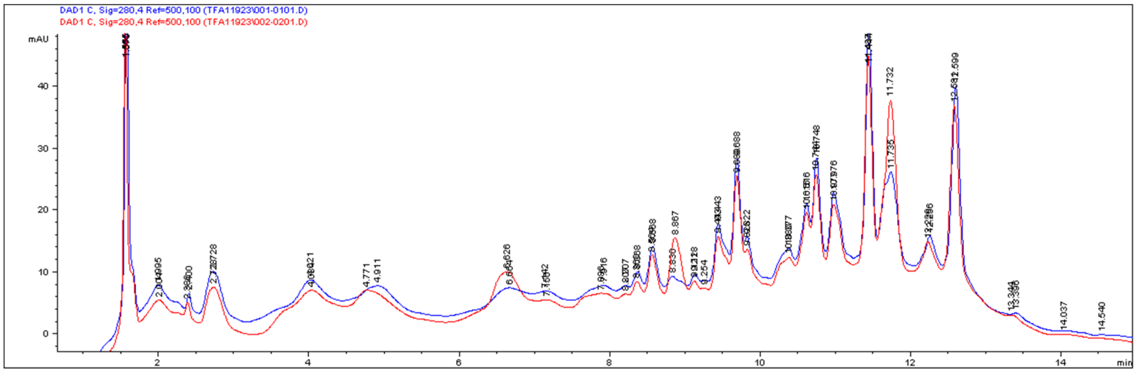

Figure S2. HPLC-DAD chromatogram of SbLEx: C (tr= 6.65 min) and EC (tr= 8.83 min), λ= 280 nm-blue line; chromatogram of fortified SbLEx: C (tr= 6.63 min) and EC (tr= 8.87 min)-red line.

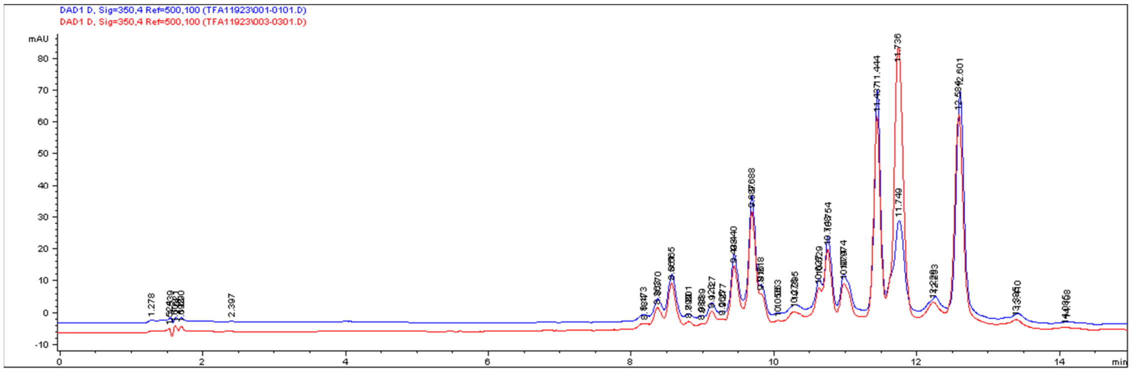

Figure S3. HPLC-DAD chromatogram of SbLEx: quercetin 3-rutinoside (tr= 11.75 min), λ= 350 nm-blue line; chromatogram of fortified SbLEx: quercetin 3-rutinoside (tr= 11.74 min)-red line.

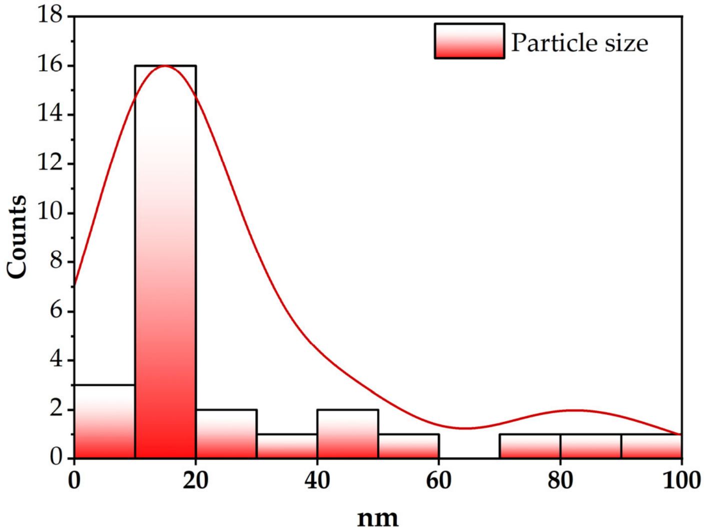

Figure S4. Particle size distribution of SeNPsSb from the TEM image (Figure 2) analysis of diameters with ImageJ.

Table S2. EDX analysis of SeNPsSb

Element	Weight %	Atomic %	Uncert. %
C(K)	86.31%	92.89%	0.24
O(K)	7.22%	5.84%	0.15
Cu(K)	5.44%	1.10%	0.05
Se(K)	1.03%	0.17%	0.02
Total	100.00%	100.00%	

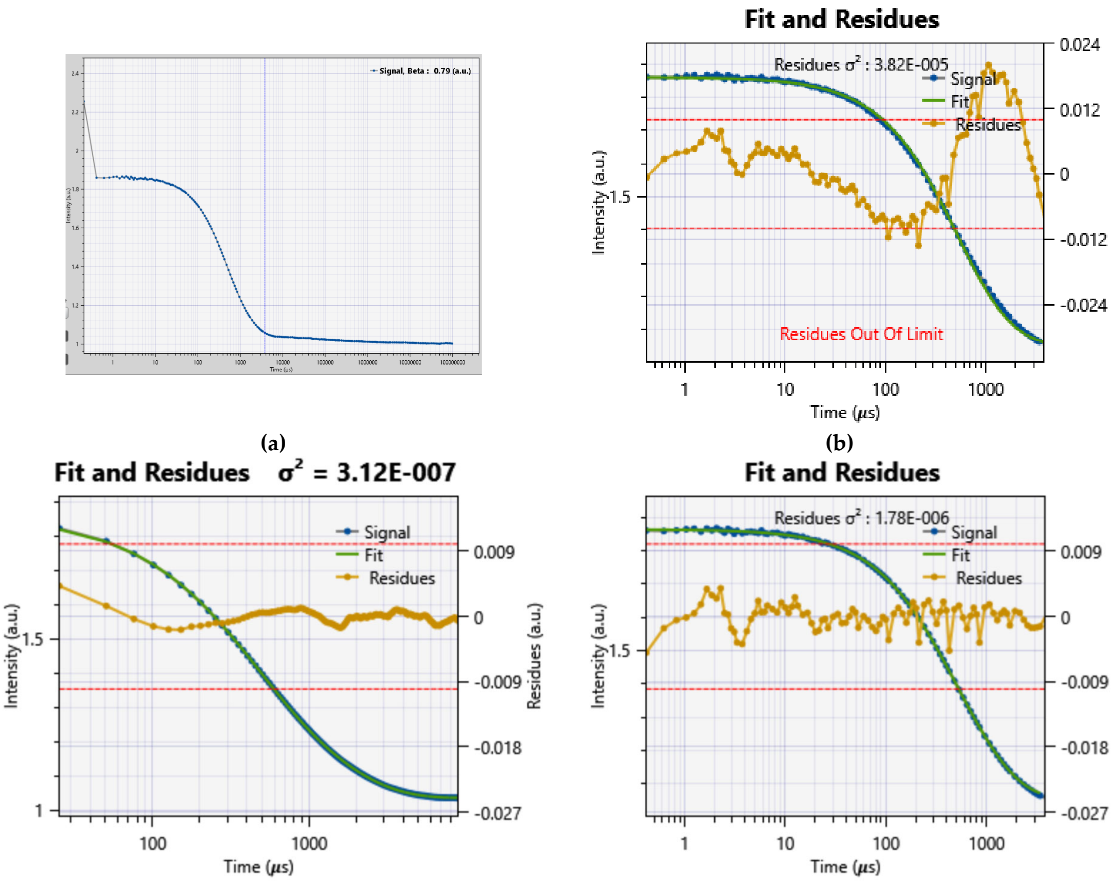

(c) (d)

Figure S5. DLS analysis: (**a**) autocorrelation function for SeNPsSb; (**b**) simulation of autocorrelation function for Cumulants method; (**c**) simulation of autocorrelation function for Pade Laplace (PL) method; (**d**) simulation of autocorrelation function for SBL method.

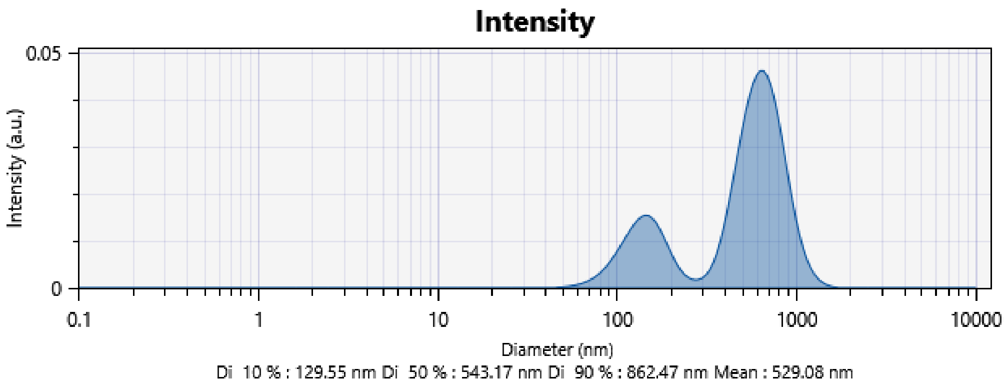

(a)

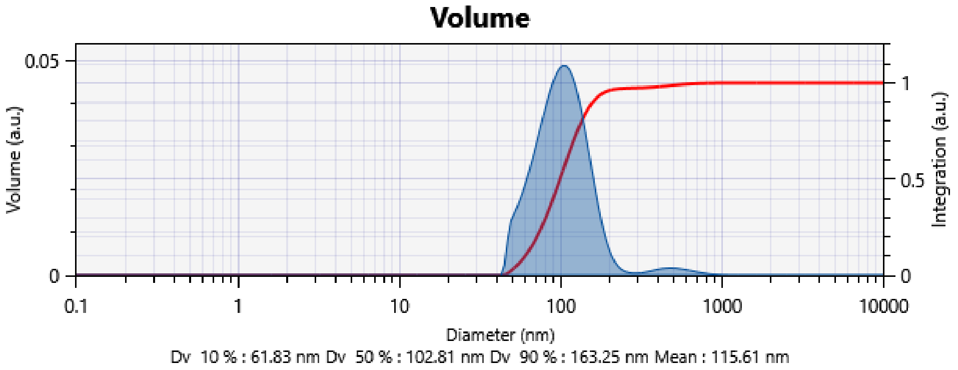

(b)

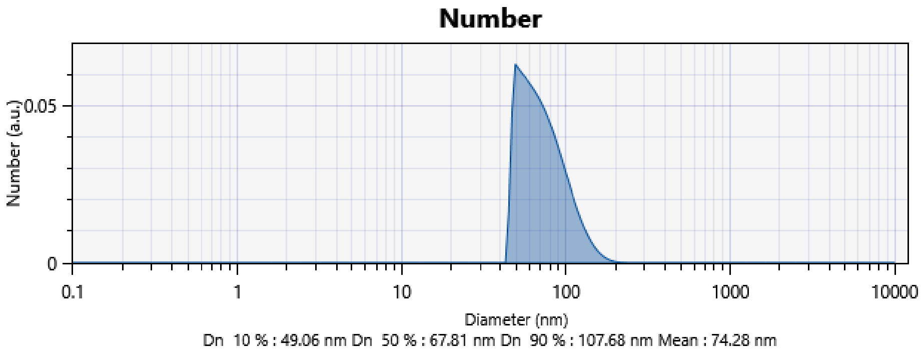

(c)

Figure S6. DLS analysis of SeNPsSb: (**a**) Intensity; (**b**) Volume; (**c**) Number.

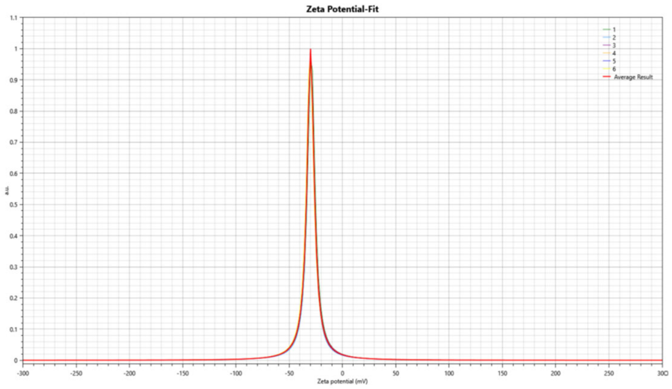

Figure S7. Zeta potential analysis of SeNPsSb.

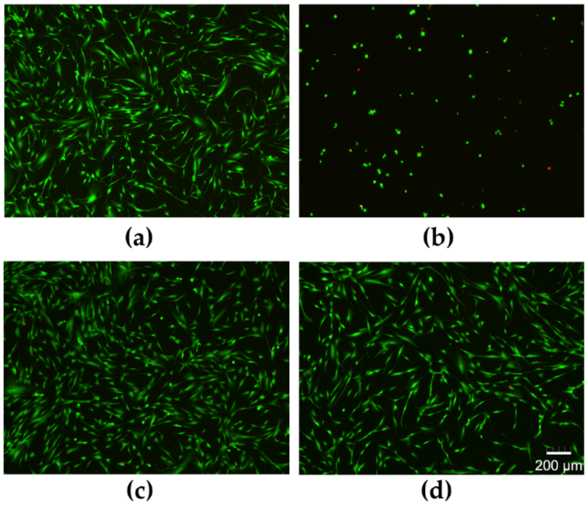

Figure S8. Cytocompatibility of SeNPsSb: (**a**)–(**d**) LIVE/DEAD assay (green fluorescence indicates live cells, red fluorescence indicates dead cells): (**a**) untreated cells; (C−, negative cytotoxicity control); (**b**) cells treated with 7.5% DMSO (C+, positive cytotoxicity control); (**c**) cells treated with 0.5 µg/mL SeNPsSb; (**d**) cells treated with 2.5 µg/mL SeNPsSb.

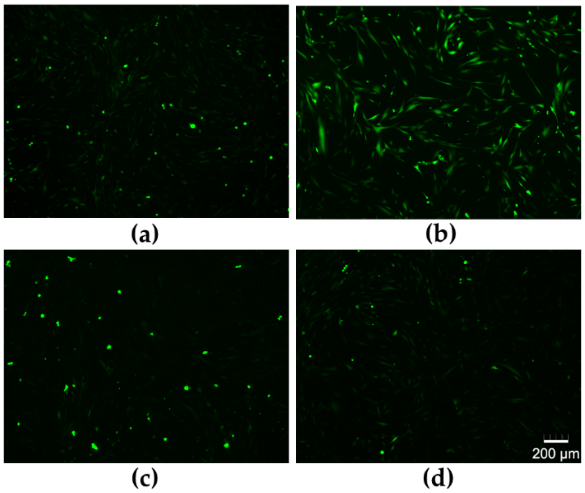

Figure S9. In vitro antioxidant activity of SeNPsSb: (**a**)–(**d**) Fluorescence microscopy images after labeling the total intracellular ROS with H_2DCFDA (green fluorescence): (**a**) untreated cells (C−, negative control); (**b**) cells treated with 37 µM H_2O_2 (C+, positive control; ROS inducer); (d-e) HGF-1 cells incubated in the presence of ROS inducer and (**c**) 0.5 µg/mL SeNPsSb; (**d**) 2.5 µg/mL SeNPsSb.

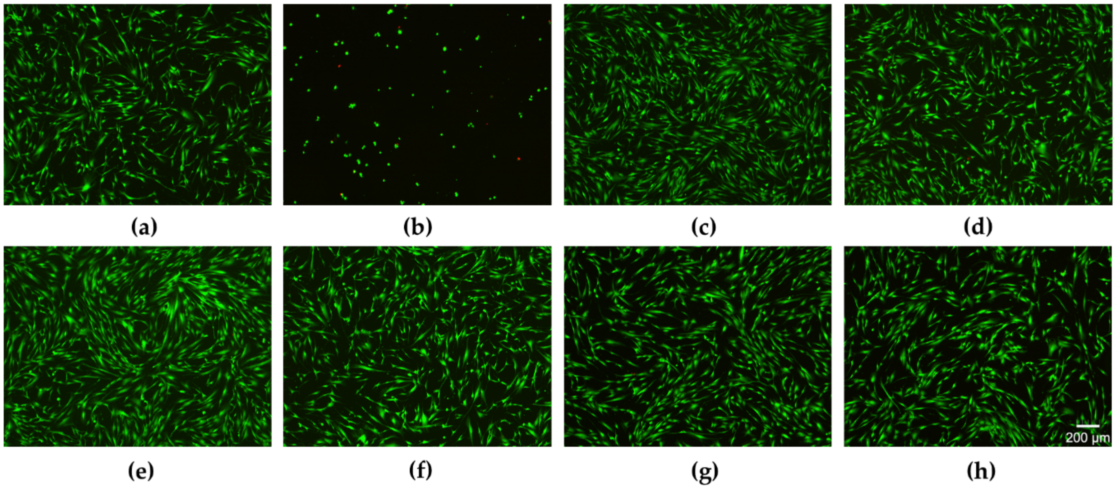

Figure S10. Cytocompatibility of Se-HNF: (**a**)–(**h**) LIVE/DEAD assay (green fluorescence indicates live cells, red fluorescence indicates dead cells): (**a**) untreated cells; (C−; negative cytotoxicity control); (**b**) cells treated with 7.5% DMSO (C+; positive cytotoxicity control); (**c**) 25 µg/mL HNF; (**d**) 1000 µg/mL HNF; (**e**) 25 µg/mL 0.5 Se-HNF; (**f**)

1000 µg/mL 0.5 Se-HNF; (**g**) 25 µg/mL 2.5 Se-HNF; (**h**) 1000 µg/mL 2.5 Se-HNF; HNF – 5% water-soluble chitosan in 0.4% never-dried bacterial nanocellulose; 0.5 Se-HNF – HNF with 0.5 µg/mL SeNPsSb; 2.5 Se-HNF – HNF with 2.5 µg/mL SeNPsSb.

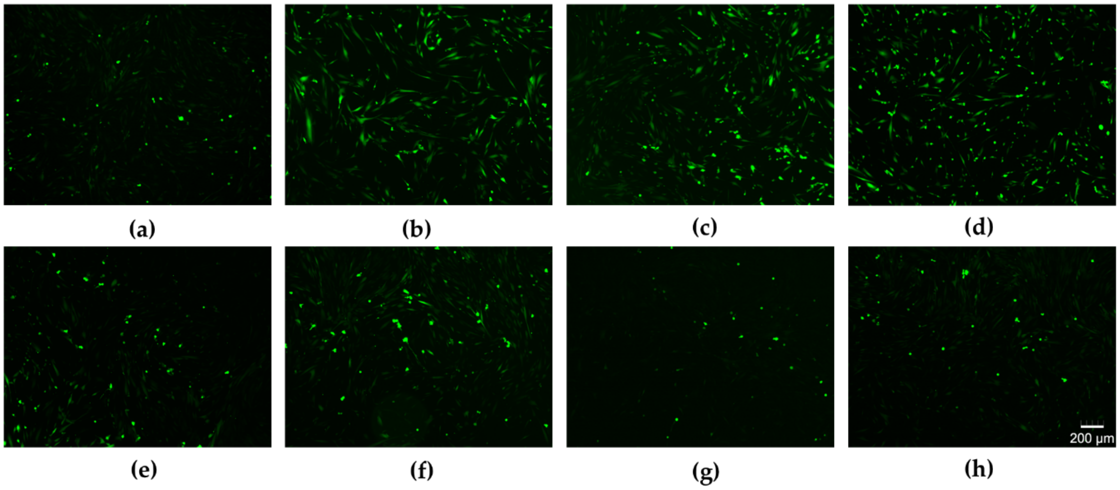

Figure S11. In vitro antioxidant activity of hydrogel formulations: (**a**)–(**h**) Fluorescence microscopy images after labeling the total intracellular ROS with H_2DCFDA (green fluorescence): (**a**) untreated cells (C−; negative control); (**b**) cells treated with 37 µM H_2O_2 (C+; positive control; ROS inducer); (e-i) HGF-1 cells incubated in the presence of ROS inducer and (**c**) 25 µg/mL HNF; (**d**) 1000 µg/mL HNF; (**e**) 25 µg/mL 0.5 Se-HNF; (**f**) 1000 µg/mL 0.5 Se-HNF; (**g**) 25 µg/mL 2.5 Se-HNF; (**h**) 1000 µg/mL 2.5 Se-HNF; HNF – 5% water-soluble chitosan in 0.4% never-dried bacterial nanocellulose; 0.5 Se-HNF – HNF with 0.5 µg/mL SeNPsSb; 2.5 Se-HNF – HNF with 2.5 µg/mL SeNPsSb.

Table S3. The inhibition zone 24 h after SeNPsSb treatment.

**SeNPsSb (mg/mL)**	***B. cereus***	***E. faecalis***	***S. aureus***	***C. albicans***
**0.05**<br>**0.1**<br>**0.2**	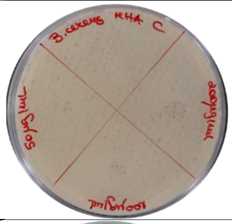	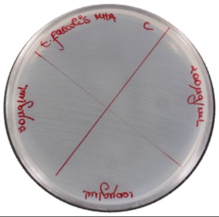	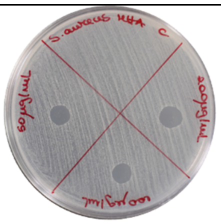	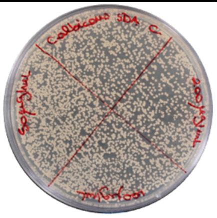
**0.5**<br>**1**<br>**2**	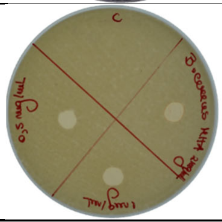	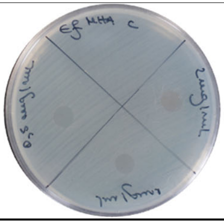	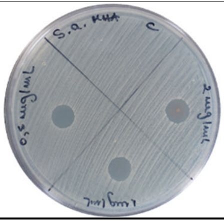	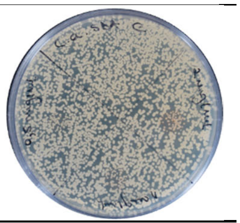

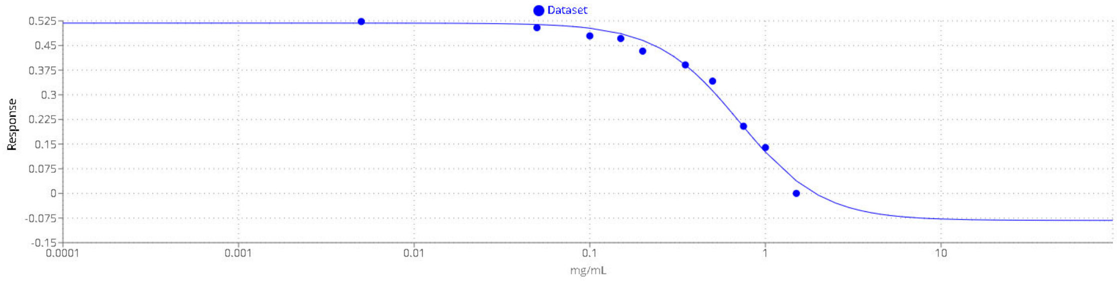

(a)

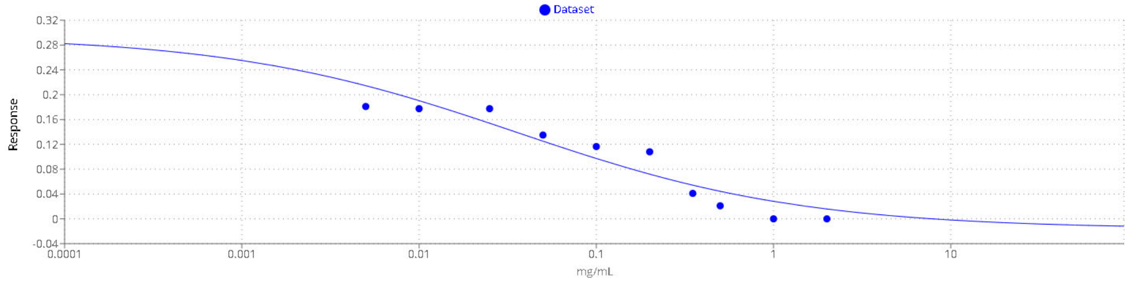

(b)

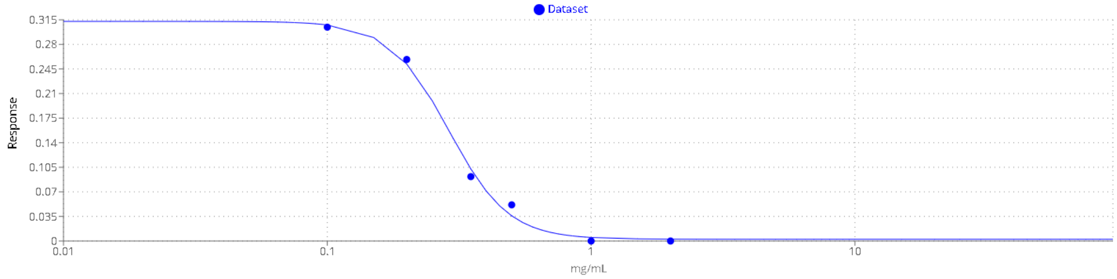

(c)

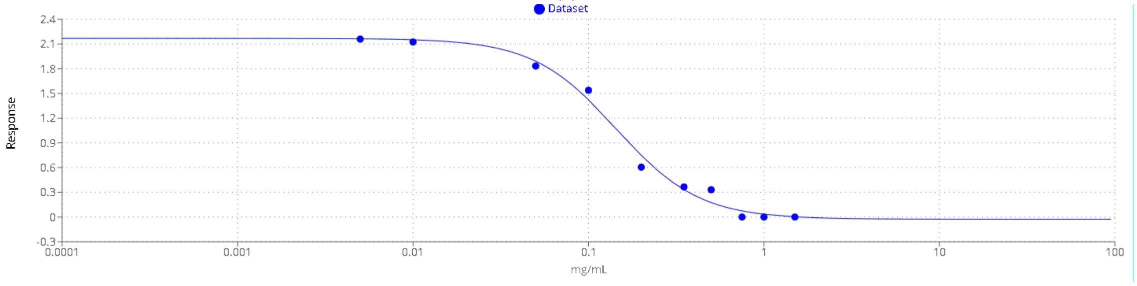

(d)

Figure S12. IC50 plot: (**a**) *B. cereus*; (**b**) *E. faecalis*; (**c**) *S. aureus*; (**d)** *C. albicans*.
